# Comparative Genomics of Plant Fungal Pathogens: The *Ustilago*-*Sporisorium* Paradigm

**DOI:** 10.1371/journal.ppat.1004218

**Published:** 2014-07-03

**Authors:** Theresa Wollenberg, Jan Schirawski

**Affiliations:** RWTH Aachen University, Microbial Genetics, Institute of Applied Microbiology, Aachen Biology and Biotechnology, Aachen, Germany; The University of North Carolina at Chapel Hill, United States of America

The closely related smut fungi *Ustilago maydis*, *U. hordei*, and *Sporisorium reilianum* f. sp. *zeae* are facultatively biotrophic basidiomycetes that occur ubiquitously. Teliospores germinate to produce sporidia of different mating type that grow saprophytically and multiply mitotically by budding [1]. For mass proliferation and sexual genetic exchange, successful colonization of economically important crop plants like maize, barley, and oats is a prerequisite. Mating of compatible haploid yeast cells leads to the formation of dikaryotic filaments that are infection competent. These filaments enter their hosts by penetration of the leaf surface [2]. Once inside the plant, filaments multiply in the affected tissue and induce spore formation in tumors near the penetration site (*U. maydis*) [3] or spread through the entire plant and form spores in inflorescences (*S. reilianum* and *U. hordei*) [4], [5]. Although presence of the fungus is clearly detected [6], defense reactions of native host plants are very limited, allowing fungal spread initially without major plant tissue damage. In fact, a living host plant is required to provide nutrients for massive fungal proliferation and successful spore formation.

## What Did We Know before Genome Sequencing?

Smut fungi have intrigued scientists for more than a century for many different reasons, among which are their host specificity, mating behavior, and ability to cause plant disease [7]. Before the molecular era, smut fungi were typically classified by identification of the plant on which symptoms were found, since smuts have a limited host range and many form spores only on a single plant species [8].

Mating behavior was one of the first things studied in smut fungi. *U. hordei* is bipolar [9], which means that germination of the diploid spore leads to haploid yeast-like meiosis products with two different mating types. In contrast, *U. maydis* and *S. reilianum* are tetrapolar, and spore germination gives rise to four different haploid yeast cell types, of which only two combinations are mating competent [10]–[12]. These early observations already led to the proposal of the presence of two independently segregating mating type loci, *a* and *b*, that each exist in several alleles. *U. maydis* possesses two *a* and more than 20 *b* alleles and was thought to represent the typical tetrapolar smut fungus. Only much later was it discovered that *S. reilianum* had three *a* alleles with two pheromone genes each [13], and even later that the occurrence of three *a* alleles might have been the earlier state during smut fungal evolution (see below) [14].

The start of the molecular era made it possible to identify pathogenicity genes. The first ones identified were the mating type genes and genes involved in signal transduction of the pheromone stimulus [15]. This research unraveled the complex signaling pathways that take place when two mating competent cells meet and form an infectious dikaryotic filament and supports the notion that mating is a prerequisite for plant infection. However, identification of bona fide virulence genes involved in the plant-fungus communication was not successful until the start of the genome sequencing era.

## What Did We Learn from Sequencing Smut Genomes?

Because of its virulence in maize and its molecular accessibility, *U. maydis* was the first smut fungus to be sequenced [3]. After sequencing by two private companies, it was also sequenced by the public sector using classical Sanger sequencing. Genome sequences of *S. reilianum* f. sp. *zeae* and *U. hordei* were assembled from Roche/454 sequencing reads, and *S. reilianum* was one of the first eukaryotes to be de novo sequenced using this technology [4]. In contrast to the *S. reilianum* genome, which could be well assembled from the sequencing reads, assembly of the *U. hordei* genome was only possible after data integration of a whole genome shotgun and a 10 kb paired-end, as well as an end-sequenced bacterial artificial chromosome (BAC) clone library [5].

*S. reilianum* and *U. maydis* have small genomes of about 20 Mb, encoding around 6,700 genes distributed on 23 chromosomes. *U. hordei* has the same number of chromosomes, but its genome is larger (26.1 Mb) and encodes more genes (7,113) [5]. This increase in genome size is not explained by the higher number of genes (since these have a smaller average size) but by a high amount of repetitive DNA [5]. The high content of repetitive DNA resulted in almost 5 Mb of small nonassembled contigs [5] and the sequence assembly problems mentioned above.

All three fungi contain a smaller amount of genes encoding cell-wall-degrading enzymes compared to necrotrophic pathogens [3]–[5], [16]. Having only a few cell-wall-degrading enzymes turned out to be a hallmark of biotrophic fungi that depend on living host tissue and thus need to avoid major damage to the plant [16]. In the *U. maydis* genome sequence, 12 gene clusters were found that encode small secreted proteins without homologs in any database and lacking enzyme-associated functional domains. This finding, combined with the knowledge that the clustered genes were highly up-regulated during plant infection, prompted a cluster-deletion study. Deletion of five of the 12 clusters resulted in an altered virulence phenotype of *U. maydis*, confirming involvement of cluster-encoded proteins in virulence [3].

## What Did We Learn from Comparing Smut Genomes?

**Figure 1:**
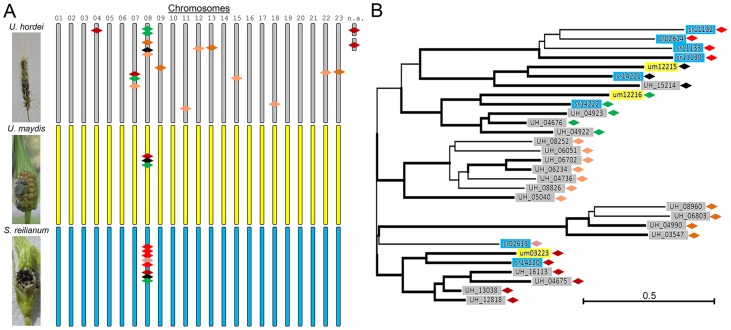
Overview of *mig1*-related genes in *U. hordei*, *U. maydis*, and *S. reilianum*. The family of *mig1*-related secreted effectors in *U. maydis* are on chromosome 8 and form a cluster of secreted proteins, whose deletion leads to hypervirulence [4]. In *S. reilianum*, the gene family is increased, but the genes are still clustered on chromosome 8 and have been identified as a divergence region [4]. In *U. hordei*, many more copies of *mig1*-related genes are present that likely have been shuffled all over the genome by TE activity [5]. (**A**) Typical symptoms of smut infection on barley (*U. hordei*) and maize (*U. maydis* and *S. reilianum*) (left panels) and schematic location of *mig1*-related genes (colored diamonds) on the 23 chromosomes of *U. hordei* (grey), *U. maydis* (yellow), and *S. reilianum* (blue) (right panels). (**B**) Tree of Mig1 proteins showing similarities of individual Mig1 proteins of the different organisms. Bold subtrees showed bootstrap support (≥50%) after 10,000 iterations. UH Mig2 was used as outgroup (not shown). Key: 01–23, chromosome number; n.a., location not assigned. Abbreviations in gene names: UH, *U. hordei*; um, *U. maydis*; sr, *S. reilianum* f. sp. *zeae*. Green: um12216 and related genes, black: um12215 and related genes, red and derivatives: um03223 and related genes—shades of red depict relatedness among the genes.

*U. maydis* and *S. reilianum* f. sp. *zeae* are both able to form spores on the same host plant, maize, but cause different symptoms (Figure 1A, left panels). Likely, fungal proteins involved in determining symptom specificity during fungal growth in planta are proteins in need of constant change to escape recognition by the plant. Therefore, these proteins are expected to show weak conservation of encoded amino-acid sequences and might thus be recognized by genome comparison. Comparison of the *U. maydis* and *S. reilianum* genomes revealed a very high similarity: about 95% of all genes occur in both organisms, and most of them are located at syntenic positions [4]. This allowed a gene-by-gene comparative analysis, which revealed 43 genomic regions containing at least three consecutive genes with predicted protein sequence identities well below average. Some of these divergence regions corresponded to the gene clusters encoding secreted proteins [4] previously identified in the genome of *U. maydis* [3]. Notably, all *U. maydis* clusters whose virulence effect was proven were reidentified as divergence regions, while three of the *U. maydis* clusters whose deletion did not affect virulence were not. Six newly found divergence regions were deleted in *U. maydis*, and four of them affected virulence [4]. These results confirmed that virulence factors can be efficiently identified using a comparison approach of related pathogens.

The largest divergence region between *U. maydis* and *S. reilianum* (cluster 19A) is located on chromosome 19 and has been shown to contain symptom specificity determinants. Deletion of the 44-kb cluster 19A region in *U. maydis* led to loss of typical *U. maydis*-specific symptoms. Deletion mutants were unable to induce anthocyanin formation and did not induce tumors on leaves. Dissection of the cluster led to identification of one factor (Tin2) as responsible for induction of anthocyanin and nine factors (Tin1-1, Tin1-2, Tin1-3, Tin1-4, Tin1-5, Tin2, Tin3, Tin4, and Tin5) together responsible for tumor induction of *U. maydis* on leaves [17]. Dissection of the complete 59 kb cluster 19A region in *S. reilianum* resulted in identification of a factor (Sad1) that enables *S. reilianum* to suppress apical dominance in infected maize plants (H. Ghareeb, F. Drechsler, C. Löfke, T. Teichmann, J. Schirawski, unpublished). Affected plants develop more female inflorescences due to outgrowth of subapical ears [18], which increases the number of sites for fungal spore formation. Interestingly, Sad1 also increases inflorescence branching when expressed heterologously in transgenic *Arabidopsis thaliana* plants, indicating that it functions via a mechanism conserved between maize and *A. thaliana* (H. Ghareeb, F. Drechsler, C. Löfke, T. Teichmann, J. Schirawski, unpublished).

## What Did We Learn about the Evolution of Smuts?

The major chromosomal differences between *S. reilianum*, *U. maydis*, and *U. hordei* can be explained by two independent chromosome rearrangements that happened during speciation and separated the most recent common ancestor with a genome organization as in *S. reilianum* from the *U. maydis* and the *U. hordei* lineages. In the *U. hordei* lineage, the chromosome rearrangement had a profound effect on fungal biology because it placed the before independently segregating *a* and *b* mating type loci on the same chromosome, introducing a physical linkage and forcing a bipolar mating behavior on *U. hordei* [5]. Accumulation of repetitive elements in the intervening regions between the *a* and *b* part of the mating locus of *U. hordei* may have led to suppression of recombination [5], [19], which represents a step towards evolution of a sex chromosome.

The most common ancestor of the three fungi was likely tetrapolar and had several *b* and three different *a* alleles. The number of *a* and *b* alleles that were retained in *U. hordei* diminished to two combinations through chromosomal linkage. In the tetrapolar *U. maydis* lineage, one *a* allele got lost (remnants of a second pheromone gene can still be found in the *a1* locus of *U. maydis*), while the number of *b* alleles increased by mutation and intra-allelic recombination events [20]. In support of this scenario, a recent investigation of mating factor distribution in smut fungi revealed a high prevalence of the third *a* allele in other smut fungi distributed along the smut fungal tree [14]. In addition to the one major chromosomal rearrangement event, transposable elements (TEs) and repetitive sequences have spread in the *U. hordei* lineage. This led to shuffling within the *U. hordei* genome that shows small regions of conserved gene order placed at rearranged chromosomal locations [5]. TE activity also seems to have contributed to distribution of duplicated effector genes. For example, 19 genes related to the *U. maydis* avirulence effector *mig1* (*maize-induced gene 1*) exist in *U. hordei*, and they are distributed over at least 11 chromosomes, while in *U. maydis* three and in *S. reilianum* eight *mig1*-related genes lie clustered solely on chromosome 8 (Figure 1).

*U. hordei* is capable of RNA silencing and possesses enzymes necessary for RNA interference (RNAi). These enzymes are also present in *S. reilianum* and therefore were likely also present in their common ancestor. However, the genes for RNAi-associated enzymes are lacking in *U. maydis*. They seem to have been cleanly excised from the genome by the efficient recombination system present in *U. maydis* [4]. One explanation for the necessity to rid the genome of RNAi components may be the occurrence of killer viruses in *U. maydis*. These provide a growth advantage to *U. maydis* cells due to virally encoded killer proteins toxic to other yeasts [21]. Since the viruses carry genomic RNA, RNAi would likely silence their beneficial effects on spread of *U. maydis*.

## Will More Sequencing Be Necessary?

Absolutely. As outlined above, a lot can be learned from sequencing and comparing smut fungal genomes. In addition, sequencing of smut fungal transcriptomes at different stages of plant colonization will tell us when or in which tissues virulence effectors are expressed, which will help in the identification of effector targets in the host plant. With each new genome and transcriptome sequence at hand, the prediction of which factors are responsible for the colonization of particular niches (e.g., host plant or host tissue) will become more precise. At the moment it is possible to compare the effector proteins of *U. maydis*, *S. reilianum* f. sp. *zeae*, and *U. hordei* [5] and generate lists with effectors conserved among all three species (which would be expected to have a general role in virulence) and those that are conserved only in *U. maydis* and *S. reilianum* (and would thus be involved in colonization of maize rather than barley). The problem with the current lists is that they are too long to experimentally verify involvement of the target effectors in adaptation to a particular host and that they likely still include many “false positives”, which decreases the chance of experimental validation. Therefore, we need more genome and transcriptome sequences of closely related fungi colonizing different ecological niches. For example, much progress in virulence effector prediction can be expected from sequencing and comparison of the smut fungi *S. scitamineum*, a pathogen on sugarcane, *U. bromivora*, a pathogen on *Brachypodium*, or *Thecaphora thlaspeos*, a smut fungus able to infect Brassicaceae. However, the more closely related the compared fungi are, the more likely it is that genomic differences reflect host adaptation. Therefore, sequencing of the sorghum pathogen *S. reilianum* f. sp. *reilianum* and comparison to its maize-pathogenic relative *S. reilianum* f. sp. *zeae* is most promising to identify genes involved in host adaptation.
